# Influence of model assumptions about HIV disease progression after initiating or stopping treatment on estimates of infections and deaths averted by scaling up antiretroviral therapy

**DOI:** 10.1371/journal.pone.0194220

**Published:** 2018-03-19

**Authors:** Kanes Sucharitakul, Marie-Claude Boily, Dobromir Dimitrov, Kate M. Mitchell

**Affiliations:** 1 Department of Infectious Disease Epidemiology, Imperial College London, London, United Kingdom; 2 Vaccine and Infectious Disease Division, Fred Hutchinson Cancer Research Center, Seattle, United States of America; British Columbia Centre for Excellence in HIV/AIDS, CANADA

## Abstract

**Background:**

Many mathematical models have investigated the population-level impact of expanding antiretroviral therapy (ART), using different assumptions about HIV disease progression on ART and among ART dropouts. We evaluated the influence of these assumptions on model projections of the number of infections and deaths prevented by expanded ART.

**Methods:**

A new dynamic model of HIV transmission among men who have sex with men (MSM) was developed, which incorporated each of four alternative assumptions about disease progression used in previous models: (A) ART slows disease progression; (B) ART halts disease progression; (C) ART reverses disease progression by increasing CD4 count; (D) ART reverses disease progression, but disease progresses rapidly once treatment is stopped. The model was independently calibrated to HIV prevalence and ART coverage data from the United States under each progression assumption in turn. New HIV infections and HIV-related deaths averted over 10 years were compared for fixed ART coverage increases.

**Results:**

Little absolute difference (<7 percentage points (pp)) in HIV infections averted over 10 years was seen between progression assumptions for the same increases in ART coverage (varied between 33% and 90%) if ART dropouts reinitiated ART at the same rate as ART-naïve MSM. Larger differences in the predicted fraction of HIV-related deaths averted were observed (up to 15pp). However, if ART dropouts could only reinitiate ART at CD4<200 cells/μl, assumption C predicted substantially larger fractions of HIV infections and deaths averted than other assumptions (up to 20pp and 37pp larger, respectively).

**Conclusion:**

Different disease progression assumptions on and post-ART interruption did not affect the fraction of HIV infections averted with expanded ART, unless ART dropouts only re-initiated ART at low CD4 counts. Different disease progression assumptions had a larger influence on the fraction of HIV-related deaths averted with expanded ART.

## Introduction

Antiretroviral therapy (ART) has been shown to drastically decrease mortality in HIV-infected individuals, increasing life expectancy to levels approaching that of the general population[1], and to effectively reduce the risk of HIV transmission by decreasing viral replication and thus viral load[2]. A landmark randomized controlled trial demonstrated that early ART (administered immediately) could reduce transmission risk by 93% in serodiscordant heterosexual couples compared with delayed ART (administered following CD4 count declines or symptom onset)[3]. In 2016, the ongoing observational PARTNER study reported no phylogenetically linked transmissions among an estimated 22,000 occasions of condomless sex in serodiscordant partnerships between men who have sex with men (MSM) when the HIV-infected partner was taking ART and virally suppressed [4]. Due to the effectiveness of ART in limiting HIV transmission within couples, there has been interest in whether the scale-up of ART use could reduce population-level HIV incidence[5]. One South African study showed that an individual’s risk of acquiring HIV was lower if they lived in an area with higher ART coverage[6]. Community-randomized controlled trials aiming to estimate the population-level impact of early treatment are ongoing; one trial found no impact of early treatment on HIV incidence in rural South Africa, primarily due to low linkage of diagnosed HIV-infected people to HIV care which led to modest increases in ART coverage[7], while two other trials which have not yet been completed have reported promising increases in ART coverage in the intervention arm.

Mathematical modelling is a valuable tool for planning and evaluating public health interventions[8], and is particularly useful for population-level evaluations[9]. Mathematical models consistently suggest substantially reduced HIV incidence due to expanding ART access but differ in the magnitude of the impact they predict[10].

While there are many similarities between published models, they have made different assumptions about disease progression for individuals on ART and those who have dropped out of ART[5, 11–15], and the impact of this type of structural assumption is rarely investigated in modelling studies. In this paper, we investigate each of the different sets of assumptions we are aware of that have been previously used in models investigating the impact of ART expansion.

HIV disease progression among infected individuals is commonly monitored by measuring CD4 counts. Typically, CD4 counts decline rapidly over the first few months of initial HIV infection[16], and subsequently decline at a steady but slower rate in the absence of ART[17]. Adequate ART use results in reduced viral load and increasing CD4 counts [18, 19]. Following ART interruption, a rapid decline in CD4 count is seen in the first few months, followed by slower decline [20, 21]. Following ART re-initiation, CD4 counts increase again, following a pattern similar to that seen in those initiating ART for the first time[20, 22].

In some models, the improved survivorship of individuals on ART is represented by a slower CD4 decline compared to ART-naives[5, 12]. In other models the disease progression of individuals on ART is determined by their CD4 count at the time of ART initiation[11, 13, 14], not explicilty representing changes in CD4 count while on ART. Some models explicitly represent the increasing CD4 counts of individuals on ART[15] while others also include an accelerated rate of CD4 decline in individuals who have dropped out of ART compared with those never on ART[13]. These assumptions will affect survival of those on ART and those who have dropped out of ART, and the duration for which dropouts are infectious. The extent to which these assumptions may influence the impact of expanded treatment on mortality and new HIV infections has not been systematically assessed, despite their potential influence on model predictions and public health decisions. These previous models have typically been calibrated to HIV prevalence and ART coverage, and used to estimate the impact of expanded ART on new HIV infections [5, 11–15], and, less frequently, on mortality[5, 15].Empirical estimates of rates of ART re-initiation among ART dropouts are very scarce since they are often not engaged in HIV care. It has previously been shown that the rate of ART re-initiation used in models may influence model predictions of intervention impact[23].

In this study, we simulated HIV transmission among MSM in the United States to explore how different modeling assumptions about HIV progression on and post-ART interruption used in published models influence predictions of the population-level impact of expanding ART. MSM make up approximately 2% of the US population[24], but accounted for 63% of new HIV infections in 2010[25]. Only 33% of HIV-infected MSM were estimated to be on ART in 2010[26, 27]. We compared a model of HIV transmission and treatment integrating each of four main progression assumptions in turn, and independently fitted to HIV prevalence and ART coverage among MSM in the US, and investigated the predicted impact of an intervention achieving fixed increases in ART coverage above baseline over a period of 10 years. We conducted sensitivity analysis to assess whether differences in predicted impact between different progression assumptions depend upon other factors, including ART re-initiation rates.

## Methods

### Model

We developed a new deterministic compartmental model of HIV transmission and treatment amongst US MSM, which integrated each of the four disease progression assumptions outlined in the introduction in turn, to enable us to compare results using these different assumptions[5, 11–15].

#### Shared elements between progression assumptions

The model has the same compartmental structure for all four progression assumptions, dividing men by infection and ART status. All new individuals entering the model join the susceptible compartment (S) at a constant rate *μ*•N_0_ (N_0_ is initial total population size). Individuals leave all model compartments at a per-capita rate *μ*, representing non-HIV-related death or cessation of sexual activity.

The per-capita force of HIV infection *λ*(t) takes into account the number, HIV status, stage, and ART status of sexual partners.

**Fig 1:**
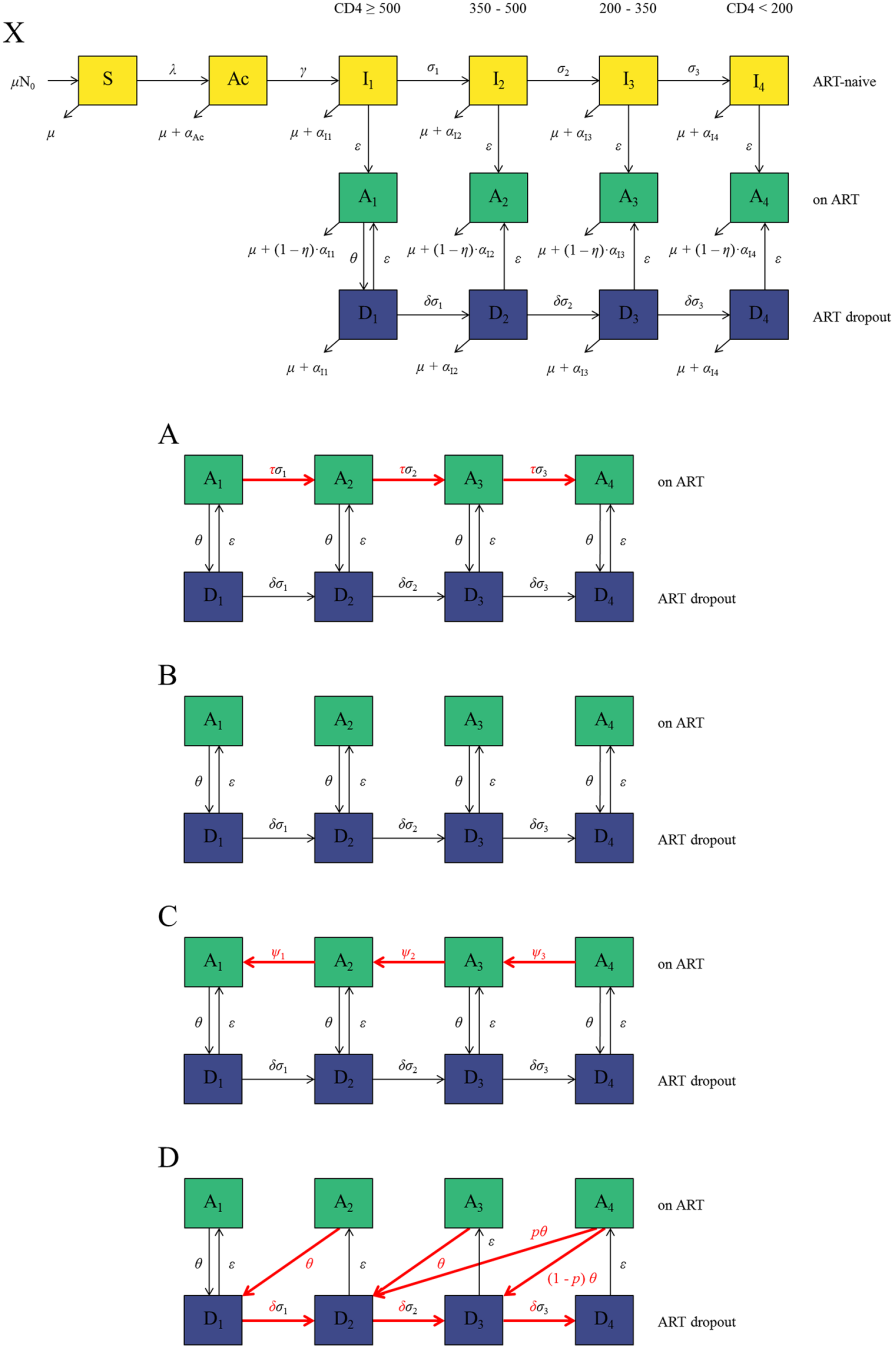
Model diagrams. **X)** General model structure showing only what is consistent across all progression assumptions, A, B, C, and D. The following model diagrams show only the ART compartments (A_i_) and ART dropout compartments (D_i_) and do not show mortality. Key differences are highlighted in red. **A)** *Progression assumption A*: ART reduces disease progression rate (*σ*_i_) by a factor ***τ*** while ART dropouts progress at the same rate as ART-naive individuals (*δ* = 1). **B)** *Progression assumption B*: There is no movement between ART compartments; prognosis depends on CD4 at ART initiation. **C)** *Progression assumption C*: ART patients progress to higher CD4 categories over time at a per-capita rate *ψ*_i_ and the rest is as in progression assumption A. **D)** *Progression assumption D*: As in assumption B, there is no movement between ART compartments. However, upon dropping out of ART, individuals move to a higher CD4 category (reflecting improvement in CD4 count on ART) but then progress at an increased rate compared to ART-naive individuals (*δ*>1; reflecting the rapid CD4 decline which occurs after dropping out of ART).

Following infection, individuals progress through the acute stage (Ac), and four chronic HIV stages (I_1-4_), segregated by CD4 count (Fig 1X), at per-capita rates *γ*, *σ*_1_, *σ*_2_, and *σ*_3_. For ART-naive individuals, the HIV-related death rate (*α*_Ii_) increases with declining CD4 count. Infectiousness is increased in the acute stage (by a factor RR_Ac_) and lower CD4 count categories (by a factor RR_Ii_) compared to individuals with CD4≥350 cells/μl.

ART-naive individuals in any chronic stage may initiate ART at a per-capita rate *ε* which is constant across stages in the main analysis.

On ART, infectiousness is reduced to the same level across all CD4 compartments, with efficacy *ω* compared to ART-naïve individuals with CD4≥350 cells/μl, while HIV-related mortality is reduced with efficacy *η* relative to their corresponding ART-naive compartments. Patients on ART drop out or fail treatment at a per-capita rate *θ*, independent of CD4 count, moving to the appropriate dropout compartment (D_1-4_).

In the main analysis, ART dropouts reinitiate ART at the same rate (*ε*) as ART-naive individuals (as not much is known about ART-re-initiation behaviours) and progress through the CD4 compartments at the rate of ART-naive individuals multiplied by a factor *δ* (which differs between progression assumptions).

Below we describe the differences between the disease progression assumptions.

#### Assumption A: Slower disease progression on ART

Progression assumption A (Fig 1A) is based on models developed by Granich et al.[5] and Cori et al.[12]. Disease progression on ART is slowed by a factor *τ* compared to ART-naive individuals. Those dropping out of ART have the same or lower CD4 count than when they initiated ART. ART dropouts progress through the CD4 compartments at the same rate as ART-naive people (*δ* = 1).

#### Assumption B: No disease progression on ART

Progression assumption B (Fig 1B) is based on the Bezemer et al.[11] and Mishra et al.[14] models. There is no movement between ART compartments, so individuals dropping out of ART have the same CD4 count as when ART was initiated. ART dropouts progress through the CD4 compartments at the same rate as ART-naive people (*δ* = 1).

#### Assumption C: Increasing CD4 count on ART

Based on the “*Optima*” model[15], the increasing CD4 count of individuals on ART is explicitly modelled. ART patients move from CD4<200 cells/μl to CD4 200–350 cells/μl, to CD4 350–500 cells/μl, to CD4≥500 cells/μl at per-capita rates *ψ*_3_, *ψ*_2_, and *ψ*_1_, respectively (Fig 1C). Those dropping out of ART have the same or higher CD4 count than when they initiated therapy. ART dropouts progress through the CD4 compartments at the same rate as ART-naive people (*δ* = 1).

#### Assumption D: Increasing CD4 count/more rapid disease progression post-ART interruption

Progression assumption D is based on a model by Eaton and Hallett[13]. There is no movement between ART compartments. Upon dropping out of ART, individuals move to a higher CD4 count compartment (Fig 1D), but thereafter progress at an increased rate compared with ART-naive individuals (*δ* >1).

The model was expressed as a system of ordinary differential equations (see S1 Appendix) which were solved numerically in Berkeley Madonna version 8.3.18 using a 4^th^ order Runge-Kutta method with fixed step-size of 0.02 years.

### Parameterization & fitting data

Biological parameters, including disease progression rates, relative infectivity by infection stage, and reduction in mortality and infectiousness on ART, were drawn from published cohort studies[17, 28–32]. Numbers of sexual partners per year and condom use came from recent studies of US MSM[33, 34]. In the main analysis, ART dropout was set to 10% per year, in line with previous models[12, 13] and US data [35, 36].

In the main analysis, the relative rate of disease progression on ART in assumption A (*τ*) equals (1—*η*) as progression was tied to HIV mortality in the original models[5, 12]. For progression assumption C, the rates at which ART patients progress to higher CD4 compartments were estimated from a US cohort[18]. For progression assumption D, the proportions of those dropping out of ART moving to each CD4 category, and the relative rate of disease progression for ART dropouts vs. ART-naïve, were based on the original model[13].

HIV prevalence and ART coverage data were obtained for US MSM [26, 27].

See Tables 1 and 2 for further information on model parameters.

**Table 1:** General parameter symbols, definitions, baseline values, sensitivity analysis ranges, and sources.

General parameters—identical for progressions assumptions A, B, C, and D				
Symbol	Definition	Main parameter estimate	Range used in sensitivity analysis	Source
***γ***	Rate of progression from the acute infection stage to chronic infection with CD4 ≥ 500 (year^-1^)	4.80		[32]
***σ*_1_**	Rate of progression for ART-naive individuals from CD4 ≥ 500 to 350 ≤ CD4 < 500 (year^-1^)	1.35		[17]
***σ*_2_**	Rate of progression for ART-naive individuals from 350 ≤ CD4 < 500 to 200 ≤ CD4 < 350 (year^-1^)	0.33		[17]
***σ*_3_**	Rate of progression for ART-naive individuals from 200 ≤ CD4 < 350 to CD4 < 200 (year^-1^)	0.27		[17]
***c***	Mean number of partners per year	2.3		[33]
***κ***	Proportion of partnerships in which condoms are used	45.9%		[34]
***ν***	Efficacy of condoms in reducing transmissibility in a partnership	78%		[31, 37, 38]
***ω***	Efficacy of ART in reducing HIV transmissibility	92%	50–100%	[28]
***θ***	ART dropout rate (year^-1^)	0.10	0.05–0.20	[12, 13, 35, 36]
***μ***	Inverse of the sexual life expectancy (year^-1^)	1/ 50 = 0.02		[39]
***η***	Efficacy of ART in reducing HIV-attributable mortality	90%		[30]
***α*_Ac_**	Additional mortality attributable to HIV infection during acute infection (year^-1^)	0.00		Assumed negligible
***α*_I1_**	Additional mortality attributable to HIV infection in ART-naive individuals with CD4 ≥ 500 (year^-1^)	0.007		[29]
***α*_I2_**	Additional mortality attributable to HIV infection in ART-naive individuals with 350 ≤ CD4 < 500 (year^-1^)	0.006		[29]
***α*_I3_**	Additional mortality attributable to HIV infection in ART-naive individuals with 200 ≤ CD4 < 350 (year^-1^)	0.007		[29]
***α*_I4_**	Additional mortality attributable to HIV infection in ART-naive individuals with CD4 < 200 (year^-1^)	0.262		[29]
**RR_Ac_**	Relative infectivity of individuals in the acute phase of infection vs. chronic ART-naive CD4 ≥ 350	11.7		[32]
**RR_I3_**	Relative infectivity of infected ART-naive individuals with 200 ≤ CD4 < 350 vs. CD4 ≥ 350	1.6		[28]
**RR_I4_**	Relative infectivity of infected ART-naive individuals with CD4 < 200 vs. CD4 ≥ 350	5.0		[28]

**Table 2:** Assumption-specific parameter symbols, definitions, baseline values, and sources.

Assumption-specific parameters and variables						
Symbol	Definition	Estimate for each assumption				Source
		A	B	C	D	
***ε***	ART initiation rate for ART-naives and for dropouts (year^-1^)	0.0651	0.0641	0.0614	0.0641	Fitted
***ρ***	Transmission probability per partnership with chronically infected ART-naive individuals with CD4 ≥ 350	0.0361	0.0356	0.0343	0.0359	Fitted (range: 0.012–0.145 [40])
***τ***	Relative disease progression rate for individuals on ART compared to ART-naive	0.1	0	0	0	[5, 12, 30]
***ψ*_1_**	Rate of progression for individuals on ART from 350 ≤ CD4 < 500 to CD4 ≥ 500 (year^-1^)	0	0	0.550	0	[18]
***ψ*_2_**	Rate of progression for individuals on ART from 200 ≤ CD4 < 350 to 350 ≤ CD4 < 500 (year^-1^)	0	0	0.408	0	[18]
***ψ*_3_**	Rate of progression for individuals on ART from CD4 < 200 to 200 ≤ CD4 < 350 (year^-1^)	0	0	0.479	0	[18]
***p***	Proportion of individuals dropping out from A_4_ that move to D_2_	0	0	0	0.5	[13]
***δ***	Relative disease progression rate for ART dropouts compared to ART-naive individuals	1.0	1.0	1.0	2.0	[5, 12, 13]

### Model fitting

For each progression assumption, the model was run to equilibrium and independently fitted to the same HIV prevalence and ART coverage data (Table 3) (i.e. pre-intervention) by varying the ART uptake rate (*ε*) and per-partnership transmission probability (*ρ*) and minimizing the sum of the squared residuals using the Berkeley Madonna “Curve Fit” function. The model was not fitted to AIDS mortality data, as no data corresponding to the modelled population could be found; note that previous models were not fitted to mortality data.

**Table 3:** Model fitting data: Baseline values, sensitivity analysis ranges, and sources.

Data for model calibration for all progression assumptions			
Variable	Estimate	Sensitivity analysis	Source
Equilibrium ART coverage	33%	10–50%	[26, 27]
Equilibrium HIV prevalence	18%	10–40%	[27]

### Plan of analysis

#### Analyses without intervention

We calculated survival times (by ART status), HIV incidence and population distribution by CD4 count and ART status for each progression assumption at equilibrium (pre-intervention).

#### Main analysis: ART intervention scenarios

Using the main parameter values for each progression assumption, we increased ART coverage from its baseline equilibrium value (33%) either by increasing ART uptake (*ε*) or decreasing ART dropout (*θ*). These parameters were varied to produce final ART coverage values 10 years after the intervention started between 33% and 90%. Intervention impact was measured as percentage of new infections and HIV-related deaths averted over the first 10 years, compared to the baseline scenario where ART coverage remains at 33% over 10 years (keeping the ART uptake and dropout rates fixed at pre-intervention levels).

#### Sensitivity analysis

**Fig 2:**
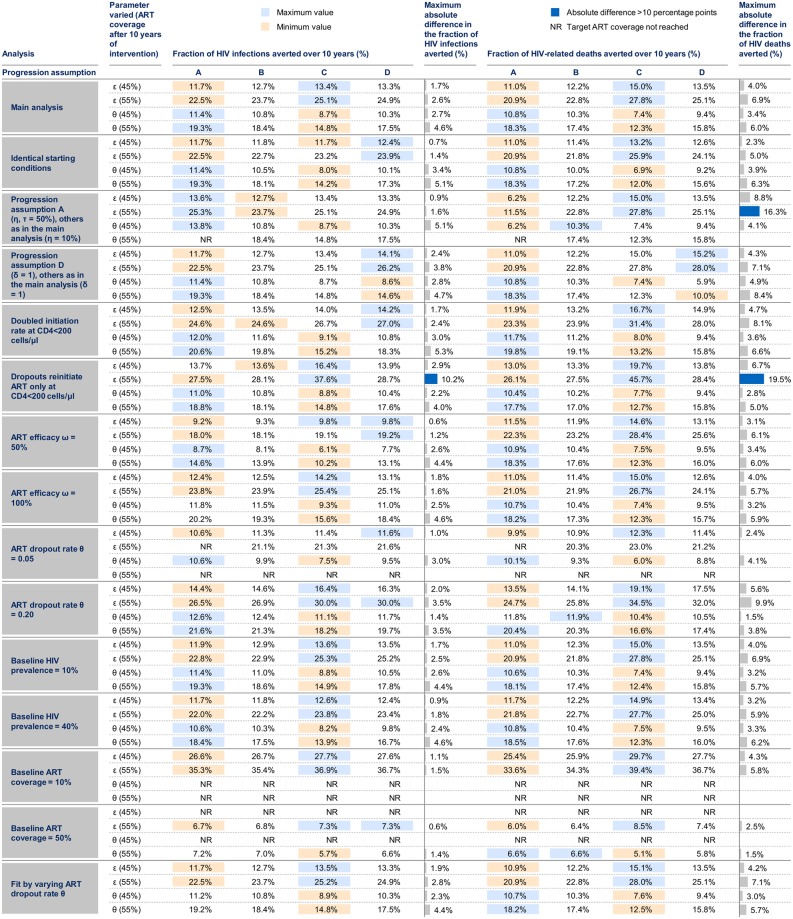
Summary of results. Each model reaches the specified ART coverage target (in brackets) 10 years after the intervention, which is achieved by either increasing the ART uptake rate (*ε*) or decreasing the ART dropout rate (*θ*). Maximum absolute differences are the differences between the minimum and maximum estimates across progression assumptions; these are only calculated when estimates are available for all 4 progression assumptions. Blue bars indicate that the absolute difference between progression assumptions in the fraction averted is greater than 10 percentage points. NR: the target ART coverage could not be reached for this progression assumption and the specified intervention.

We tested the predicted intervention impact with each progression assumption in various scenarios (Fig 2), including a version of progression assumption A with HIV-attributable mortality and disease progression rate on ART reduced by 50% (in line with the original models[12]) rather than 90%, and a version of progression assumption D with the same (rather than doubled) progression rate in ART dropouts as ART-naive individuals.

For all progression assumptions, we looked at a scenario where individuals with CD4<200 cells/μl (ART naïve and ART dropouts) initiate treatment at double the rate of higher CD4 categories, and a scenario in which ART dropouts only reinitiate ART with CD4<200 cells/μl.

We investigated the influence of varying the efficacy of ART in reducing HIV transmissibility (*ω*), baseline ART dropout rate (*θ*), baseline HIV prevalence, and baseline ART coverage. For all these analyses, the model was re-fitted to HIV prevalence and ART coverage at equilibrium by varying the transmission probability and ART uptake rate. Finally, we investigated the impact of fitting the model by varying the baseline ART dropout rate rather than the uptake rate, and the impact of using identical starting conditions in each progression assumption (rather than fitted values for transmission probability and ART uptake).

## Results

### Analyses without intervention

At equilibrium, survival of ART-naive individuals was the same for all progression assumptions (mean 9.6 years). Survival on ART was longest for progression assumption C (47.7 years) and shortest for progression assumption A (32.1 years). Survival of ART dropouts was longest for progression assumption C (8.8 years) and similar across the other 3 assumptions (6.4–6.8 years) (see S1 Table and S1 Fig). HIV prevalence was 18.0% for all models at equilibrium, in agreement with the data used for fitting[27]. HIV incidence at equilibrium was lower for assumption C (1.49/100 person-years) than for the other assumptions (1.65–1.69/100 person-years), differing by up to 13% between assumptions, but for all assumptions the estimated HIV incidence fell within the 95% CI of a national estimate of HIV incidence among MSM in 2010–2011 (1.87 per 100 person-years, 95% CI 1.04–3.38 per 100 person-years[41]). HIV-related mortality among HIV-infected MSM at equilibrium was also lower for assumption C (4.79/100 person-years) than for other assumptions (5.49–5.67 per 100 person-years), differing by up to 18% between assumptions. Similarly, HIV-related mortality among those on ART was lowest for assumption C (0.20/100 person-years) varying between 0.71–0.89/100 person-years for assumptions A, B and D, which is broadly consistent with AIDS-related mortality rates from a combined analysis of European and North American ART cohorts of around 0.6/100 person-years[42].

For assumption C, a greater proportion of the HIV-infected population had CD4>500 cells/μl than for other progression assumptions (31% vs. 8–10%). There was also a greater proportion of ART dropouts in assumption C than in assumptions A, B, and D (21% vs. 15–16%) (see S2 Fig).

### Main analysis: ART intervention scenarios

From an initial ART coverage of 33%, with 10% ART dropout per year, the maximum target ART coverage of 90% could always be reached within 10 years by increasing the ART uptake rate alone, to a rate of 1 year^-1^ for each progression assumption. However, it was not possible to reach the 90% target by decreasing the ART dropout rate alone. Reducing ART dropout from 10% year^-1^ to 0% increased ART coverage to a maximum of 56–57% within 10 years, across all progression assumptions.

Despite differences in model structure and parameter values, each assumption produced similar trends in ART coverage over time and concomitant reductions in HIV prevalence, with less than 1 percentage point (pp) difference in predicted HIV prevalence between assumptions for any given increase in ART coverage (see S3 Fig). Similar changes in the distribution of individuals in each ART stage were seen across assumptions as ART coverage increased (see S4 Fig).

**Fig 3:**
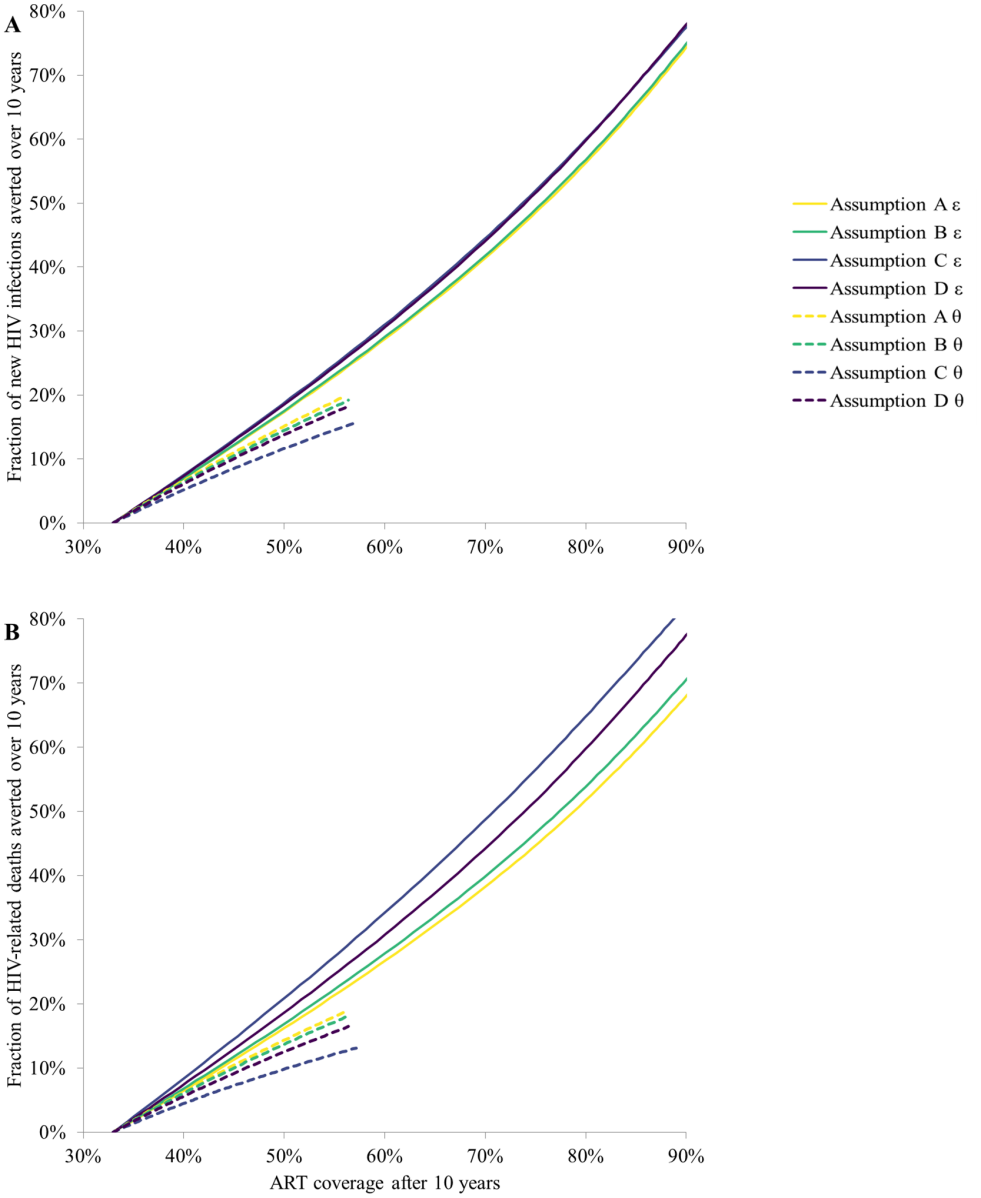
Projections from progression assumptions A-D. **A)** The fraction of HIV infections averted and **B)** The fraction of HIV-related deaths averted over the 10 year period when increasing ART uptake rate (*ε*, solid lines) or decreasing ART dropout rate (***θ***, dashed lines) to obtain final ART coverage shown.

The predicted fraction of infections averted differed by at most 5pp between progression assumptions, for ART coverage up to 90%, whether ART coverage was increased by increasing ART uptake rates or decreasing ART dropout rates (Fig 3A). For the same level of final ART coverage, decreasing ART dropout rates produced a lower estimate of ART impact and created larger differences between assumptions compared to increasing ART uptake rates.

Different progression assumptions had a larger influence on the predicted fraction of HIV-deaths averted than on the fraction of infections averted, with up to 14.2pp difference between assumptions when 90% ART coverage was achieved (Fig 3B).

### Sensitivity analysis

**Fig 4:**
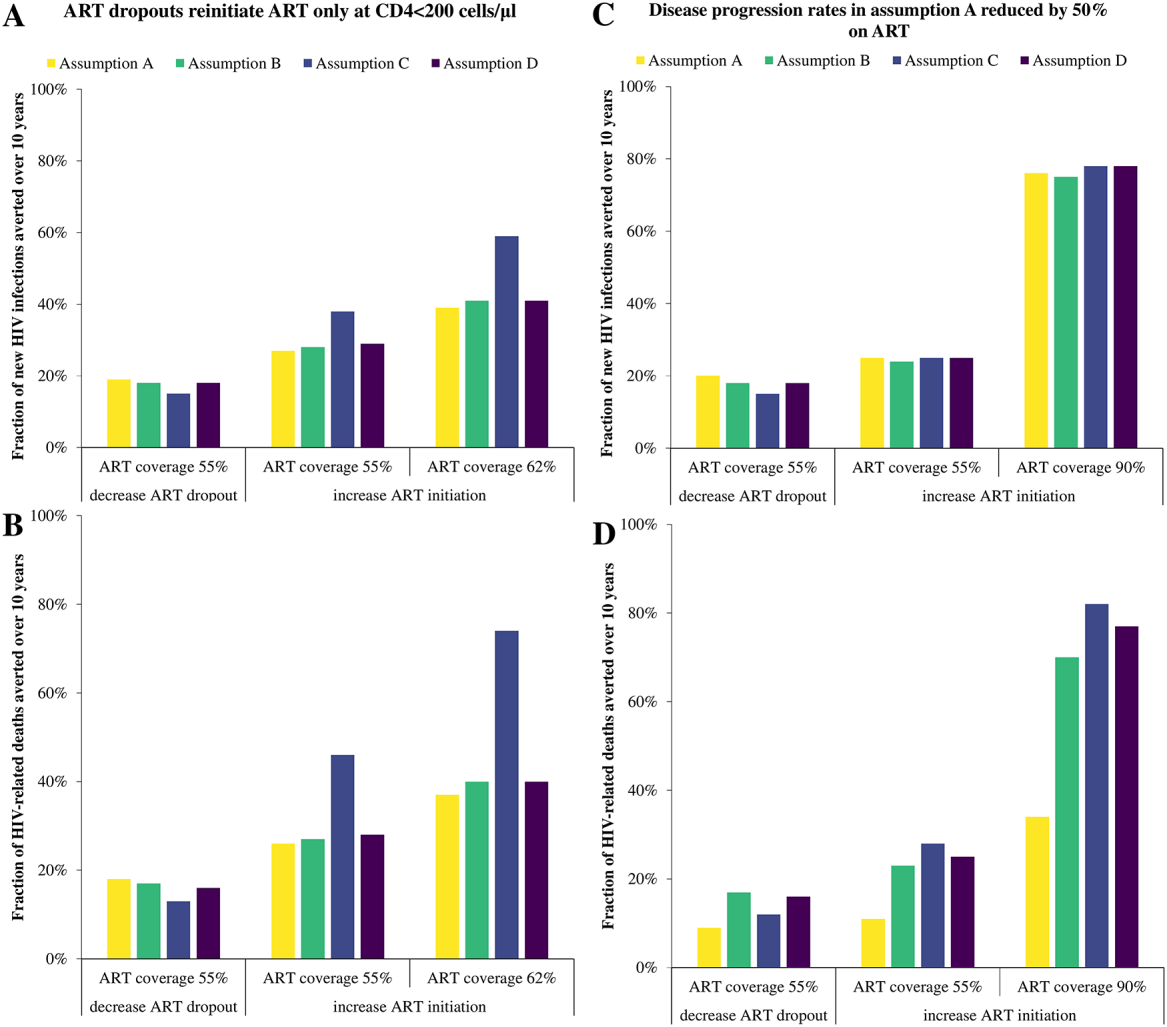
Sensitivity analysis—ART dropouts reinitiate ART only at CD4<200 cells/μl. **A)** The fraction of HIV infections averted and **B)** The fraction of HIV-related deaths averted over the 10 year period when ART initiation rate is increased or ART dropout rate is decreased to achieve a final ART coverage of 55% or 62%. HIV-attributable mortality and disease progression rates in assumption A reduced by 50% on ART vs. off ART instead of 90%: **C)** The fraction of HIV infections averted and **D)** The fraction of HIV-related deaths averted over the 10 year period when ART initiation rate is increased or ART dropout rate is decreased to achieve a final ART coverage of 55% or 90%.

Allowing ART dropouts to reinitiate treatment only at CD4<200 cells/μl led to substantial differences between assumptions (Fig 4). If ART coverage was increased through higher ART uptake rates, assumption C predicted a much greater fraction of infections and HIV-related deaths averted than the other assumptions for the same ART coverage (e.g. 10pp difference in infections averted with 55% ART coverage). Smaller variations in the fractions of infections and HIV-deaths averted were predicted for this scenario when ART coverage was increased by reducing ART dropout. Allowing ART dropouts to reinitiate treatment only at CD4<200 cells/μl affected the distribution of HIV-infected persons within the model compared to the main analysis: for assumption C, individuals stopping treatment were sequestered to the higher CD4 count ART dropout compartments as ART uptake was increased (see S5 Fig).

If 50% reduction of the HIV-attributable mortality and disease progression rates was assumed on ART in assumption A, the final HIV prevalence was lower for A compared to B-D (see S6 Fig), and the mean survival time of individuals who (re-)initiate ART for life decreased from 32.1 years to 12.1 years, while the mean survival time of ART dropouts who never reinitiate treatment decreased by 0.4 years (6% decrease). This version of assumption A predicted fewer HIV-related deaths would be averted than for other assumptions with differences between assumptions reaching 48pp if ART coverage was increased by higher ART uptake rates, but did not give substantial differences in predicted infections averted (Fig 4).

Differences between the progression assumptions remained similar in magnitude to those seen in our main analysis (up to 7pp for infections and up to 20pp for deaths averted) in the following alternative scenarios: when ART initiation rate was doubled for CD4<200 cells/μl; for different levels of ART efficacy (50–100%), baseline ART dropout rates (5–20% per year), baseline HIV prevalence (10–40%), and baseline ART coverage (10–50%); when CD4 decline for ART dropouts and ART naive MSM in assumption D were equal; when identical starting conditions were used for each progression assumption; when the model was fit by varying the ART dropout rate (instead of ART uptake rate).

## Discussion

Our study shows that in many cases, after fitting to the same HIV prevalence, assumptions about HIV disease progression during and post-ART interruption do not substantially influence the predicted impact of expanding ART coverage on HIV infections averted (up to 7 percentage point difference in the fraction of infections averted in most scenarios), except when ART dropouts reinitiate ART only at low CD4 counts (representing re-initiation due to symptoms), where substantial differences were seen. We also found that these different assumptions can substantially influence the estimated impact of expanded treatment on HIV-related deaths (up to 20pp difference between assumptions for the fraction of HIV deaths averted in many scenarios, 37pp difference if ART dropouts only reinitiate ART at low CD4 counts).

Our analyses suggest that models with slowed disease progression on ART (assumption A, following Granich et al.[5]) could give substantially smaller estimates of the impact of expanded ART on deaths if ART is only assumed to slow disease progression 2-fold, as has frequently been assumed[5, 12]. A previous analysis of this model type found little effect of the magnitude of this slowing on cumulative HIV incidence[12], in agreement with our findings, but did not look at number of deaths.

We found that models with increasing CD4 counts on ART (assumption C, e.g.[15]) could give substantially higher estimates than other progression assumptions of the impact of increased ART initiation rates on both infections and deaths averted if ART dropouts were assumed to only re-initiate ART at low CD4 levels. Under this assumption, more ART dropouts had high CD4 counts, so to reach the same ART coverage as the other assumptions a greater proportion of those with CD4<200 cells/μl must be treated. Since these individuals are more infectious and at higher risk of HIV-related death, this increases the impact.

Models assuming that CD4 count upon ART dropout is similar to CD4 count at ART initiation (assumption B, e.g.[14]) show similar results to models including both increasing CD4 counts on ART and more rapid CD4 decline following ART dropout (assumption D, e.g.[13]). This latter model comes closest to representing what is known about true CD4 dynamics on and post-treatment[17, 18, 20, 21], although long-term post-ART interruption data are scarce.

In a model comparison study for South Africa, differences between models in the predicted impact of increasing ART coverage on HIV incidence remained even when 100% treatment retention was assumed[10], in apparent contradiction to our results. Two out of 12 models in that analysis assumed lower rates of ART re-initiation among ART dropouts with high CD4 counts, but disease progression in those two models were similar to our assumptions B and D (with no sequestration of ART dropouts into high CD4 compartments). In light of our analysis, we would not expect differences in disease progression to account for the observed differences between these models; other differences between models in the South Africa study are likely to be responsible for these variations, such as calibration to different data or differences in model sexual mixing assumptions.

A number of recently published models, including several used to inform changes to the World Health Organization HIV treatment guidelines[13, 14, 23, 43], assumed lower ART initiation rates at high CD4 counts for ART dropouts, so it is important to note the difference that disease progression assumptions can make in this case. In this scenario, if we assume that assumption D (CD4 increases on ART, more rapid CD4 decline post-ART interruption) is the most realistic assumption, then models using assumption C (CD4 increases on ART without rapid CD4 decline post-ART interruption), are expected to overestimate the impact of expanded ART on both infections and deaths averted.

Differences in the predicted impact of expanded ART on deaths will have a large influence on estimates of Disability-Adjusted Life Years (DALYs) gained, a metric frequently used for cost-effectiveness analyses[43, 44]. Some of the differences we saw in impact on deaths may occur because we did not fit our models to HIV deaths (in line with previous modelling studies), whereas predictions of the impact on infections may have varied less because we fitted our models to HIV prevalence. However, this is unlikely to fully explain the large differences seen in impact on mortality when ART dropouts only re-initiated ART with lower CD4.

Simplifications of the original models needed to fit into our framework have caused the progression assumptions to differ slightly from their original published versions. Differences such as the (lack of) modelling of the care cascade, or of multiple ART stages, and using the same CD4-specific HIV-related mortality rates on ART in all progression assumptions may mean that our assumptions do not fully capture the nuances of the models they are based on [5, 11–15]. However, these differences are unlikely to substantially affect our conclusions, as more detailed representations of the care cascade or ART stages are unlikely to greatly affect the distribution of CD4 levels among those on ART or dropping out of ART, and HIV-related mortality rates at CD4 counts above 200 cells/μl are generally much smaller (by 2 orders of magnitude) than rates of movement between CD4 stages, and so again unlikely to greatly affect CD4 distributions, which are the main driver of our results. Synergistic effects between ART uptake and retention have been ignored here, as each was varied separately to increase ART coverage; Eaton et al. noted that in several of the models they tested the impact of increasing access to ART increases more rapidly with higher retention[10]. Our results apply to the first 10 years of an expanded ART intervention, but longer-term variation was not explored. We have not fully incorporated parameter uncertainty into our results, an issue noted in previous model comparison studies[10], although we have investigated the sensitivity of our results to selected key parameters, finding that they are robust to different values for ART efficacy and for ART initiation and dropout rates. It was difficult to completely separate differences in model structure from differences in parameterization in this study. However, we fitted the models in ways commonly used in the literature, enabling us to compare model results under the conditions in which they are often used. Our models were not fitted to mortality data as we were unable to find suitable data to fit our models to, which means that our estimates of the impact of expanded ART upon mortality may not be accurate. However, our finding that these estimates may vary between different model progression assumptions and with different parameters is important, since previous models estimating the impact of expanded ART provision upon mortality were also not fitted to mortality data[5, 15]

Our study has a number of strengths: the first to employ a systematic approach to compare the influence of HIV progression assumptions in modelling, this study allows the different assumptions to be compared fairly so that differences in their predictions can be attributed to differences in model structure rather than confounding due to unrelated factors such as different parameter values or methods of modelling transmission. Our sensitivity analysis, which showed that our results were consistent across different HIV prevalence and ART coverage levels, suggests that these results are broadly applicable across different settings. This modelling approach is useful for interpreting differences between models, and should be useful for comparing the influence of other model assumptions upon predictions.

## Conclusions

Differences in key disease progression assumptions on and post-ART interruption made little difference when evaluating the fraction of infections averted over 10 years of an expanded ART program, except in the case where ART dropouts re-initiated ART only at low CD4 counts, which has frequently been assumed in previous modelling studies. Differences in projected impact on HIV-related deaths were more commonly observed between progression assumptions and these differences would be likely to affect estimates of cost-effectiveness which look at DALYs averted. However, these results could be to some extent due to us calibrating the model to HIV prevalence but not to AIDS deaths.

This study has identified the following data gaps: formal comparisons of rates of CD4 decline following ART dropout vs. prior to ART initiation, and data on CD4 counts at ART re-initiation for ART dropouts. These data would improve the accuracy of modelling assumptions for ART dropouts.

Future studies seeking to understand the influence of model assumptions on the predicted impact of HIV interventions should look at parameter uncertainty and the influence of which aspects of the model are fitted to data, as well as structural uncertainty.

## Supporting information

S1 Appendix Model equations. (DOCX)

S1 Table Overall HIV incidence rate per 100 person-years for each progression assumption and the mean survival times at baseline for individuals who: i) become infected with HIV but never initiate ART; ii) initiate or reinitiate ART for life; iii) drop out of ART and never reinitiate treatment. (DOCX)

S1 Fig Survival curve for each progression assumption at baseline. Survival curve for individuals who: A) become infected with HIV but never initiate ART; B) initiate or reinitiate ART for life; C) drop out of ART who never reinitiate treatment.
(TIF)

S2 Fig Initial HIV-infected population distributions from progression assumptions A-D: A) categorized by CD4 count B) categorized by ART status. (TIF)

S3 Fig Time trends across progression assumptions in: A) ART coverage when increasing ART uptake rate to reach a target ART coverage of 90% after 10 years; B) ART coverage when decreasing ART dropout rate to reach a target ART coverage of 55% after 10 years; C) HIV prevalence over time when increasing ART uptake rate to reach a target ART coverage of 90% after 10 years; D) HIV prevalence over time when decreasing ART dropout rate to reach a target ART coverage of 55% after 10 years. (TIF)

S4 Fig The constituents of the population of infected people in the main analysis, categorized by ART status, against ART coverage for: A) Assumption A; B) Assumption B; C) Assumption C; D) Assumption D, when increasing ART uptake rate (*ε*, solid lines) or decreasing ART dropout rate (*θ*, dashed lines) to obtain the final ART coverage shown. ART dropouts are further subdivided based on CD4. (TIF)

S5 Fig The constituents of the population of infected people in the sensitivity analysis where ART dropouts reinitiate ART only at CD4<200 cells/μl, categorized by ART status, against ART coverage for: A) Assumption A; B) Assumption B; C) Assumption C; D) Assumption D, when increasing ART uptake rate (*ε*, solid lines) or decreasing ART dropout rate (*θ*, dashed lines) to obtain the final ART coverage shown. ART dropouts are further subdivided based on CD4 count to represent those who are ineligible to reinitiate ART (D_1-3_) and those who are eligible (D_4_). (TIF)

S6 Fig HIV prevalence after 10 years against ART coverage after 10 years when increasing ART uptake rate (*ε*, solid lines) or decreasing ART dropout rate (*θ*, dashed lines) to obtain final ART coverage shown for: A) The main analysis; B) HIV-attributable mortality and disease progression rates in assumption A reduced by 50% on ART vs. off ART instead of 90%. (TIF)
