# Supplementary material for: Influence of model assumptions about HIV disease progression after initiating or stopping treatment on estimates of infections and deaths averted by scaling up antiretroviral therapy
Source: PLoS One. 2018 Mar 19;13(3):e0194220. doi: 10.1371/journal.pone.0194220 (PMC5858778; doi:10.1371/journal.pone.0194220)
Supplement: S1 Appendix — (DOCX) [file pone.0194220.s001.docx]

**S1 Appendix.** Model equations

1. $\frac{\mathrm{dS}}{\mathrm{dt}}$ = *μ*N_0_ – S (*λ* + *μ*)
2. $\frac{\mathrm{dAc}}{\mathrm{dt}}$ = *λ*S – Ac (*γ* + *α*_Ac_ + *μ*)
3. $\frac{dI_{1}}{\mathrm{dt}}$ = γAc – I_1_ (*σ*_1_ + *ε* + *α*_Ι1_ + *μ*)
4. $\frac{dI_{2}}{\mathrm{dt}}$ = *σ*_1_I_1_ – I_2_ (*σ*_2_ + *ε* + *α*_Ι2_ + *μ*)
5. $\frac{dI_{3}}{\mathrm{dt}}$ = σ_2_I_2_ – I_3_ (*σ*_3_ + *ε* + *α*_I3_ + *μ*)
6. $\frac{dI_{4}}{\mathrm{dt}}$ = *σ*_3_I_3_ – I_4_ (*ε* + *α*_I4_ + *μ*)
7. $\frac{dA_{1}}{\mathrm{dt}}$ = *ε*I_1_ + *ε*D_1_ + *ψ*_1_A_2_ – A_1_ (*τσ*_1_ + *θ* + (1 – *η*)•*α*_I1_ + *μ*)
8. $\frac{dA_{2}}{\mathrm{dt}}$ = *ε*I_2_ + *ε*D_2_ + *τσ*_1_A_1_ + *ψ*_2_A_3_ – A_2_ (*ψ*_1_ *+ τσ*_2_ + *θ* + (1 – *η*)•*α*_I2_ + *μ*)
9. $\frac{dA_{3}}{\mathrm{dt}}$ = *ε*I_3_ + *ε*D_3_ + *τσ*_2_A_2_ + *ψ*_3_A_4_ – A_3_ (*ψ*_2_ *+ τσ*_3_ + *θ* + (1 – *η*)•*α*_I3_ + *μ*)
10. $\frac{dA_{4}}{\mathrm{dt}}$ = *ε*I_4_ + *ε*D_4_ + *τσ*_3_A_3_ – A_4_ (*ψ*_3_ *+ θ* + (1 – *η*)•*α*_I4_ + *μ*)
11. $\frac{dD_{1}}{\mathrm{dt}}$ = *θ*A_1_ + *Δ*•*θ*A_2_ – D_1_ (*δσ*_1_ + *ε* + *α*_Ι1_ + *μ*)
12. $\frac{dD_{2}}{\mathrm{dt}}$ = (1 – *Δ*)•*θ*A_2_ + *δσ*_1_D_1_ + *Δ*•*θ*A_3_ + *Δ*•*pθ*A_4_ – D_2_ (*δσ*_2_ + *ε* + *α*_Ι2_ + *μ*)
13. $\frac{dD_{3}}{\mathrm{dt}}$ = (1 – *Δ*)•*θ*A_3_ + *δσ*_2_D_2_ + *Δ*•(1 – *p*)•*θ*A_4_ – D_3_ (*δσ*_3_ + *ε* + *α*_I3_ + *μ*)
14. $\frac{dD_{4}}{\mathrm{dt}}$ = (1 – *Δ*)•*θ*A_4_ + *δσ*_3_D_3_ – D_4_ (*ε* + *α*_I4_ + *μ*)
15. N = S + Ac + I_1_ + I_2_ + I_3_ + I_4_ + A_1_ + A_2_ + A_3_ + A_4_ + D_1_ + D_2_ + D_3_ + D_4_
16. *λ*(t) = $\frac{c}{N}$ *ρ* (1 - *κν*) (RR_Ac_Ac + I_1_ + I_2_ + RR_I3_I_3_ + RR_I4_I_4_ + (1 – *ω*) (A_1_ + A_2_ + A_3_ + A_4_) + D_1_ + D_2_ + RR_I3_D_3_ + RR_I4_D_4_)
