# Supplementary material for: Influence of model assumptions about HIV disease progression after initiating or stopping treatment on estimates of infections and deaths averted by scaling up antiretroviral therapy
Source: PLoS One. 2018 Mar 19;13(3):e0194220. doi: 10.1371/journal.pone.0194220 (PMC5858778; doi:10.1371/journal.pone.0194220)
Supplement: S1 Table — (DOCX) [file pone.0194220.s002.docx]

**S1 Table.** Overall HIV incidence rate per 100 person-years, HIV prevalence and HIV-related mortality rate per 100 person-years for each progression assumption and the mean survival times at baseline for individuals who: **i)** become infected with HIV but never initiate ART; **ii)** initiate or reinitiate ART for life; **iii)** drop out of ART and never reinitiate treatment.

|  | Mean survival times (years) | | | Incidence rate (per 100 person-years) | HIV prevalence (%) | HIV-related mortality rate (per 100 person-years) | |
| --- | --- | --- | --- | --- | --- | --- | --- |
|  | **i)** ART-naive | **ii)** On ART | **iii)** ART dropouts |  |  | All HIV+ | On ART |
| Assumption A | 9.6 | 32.1 | 6.4 | 1.69 | 18.0 | 5.67 | 0.89 |
| Assumption B | 9.6 | 40.6 | 6.8 | 1.65 | 18.0 | 5.49 | 0.71 |
| Assumption C | 9.6 | 47.7 | 8.8 | 1.49 | 18.0 | 4.79 | 0.20 |
| Assumption D | 9.6 | 40.6 | 6.4 | 1.66 | 18.0 | 5.55 | 0.71 |
