# Supplementary figures and images for: Influence of model assumptions about HIV disease progression after initiating or stopping treatment on estimates of infections and deaths averted by scaling up antiretroviral therapy
Source: PLoS One. 2018 Mar 19;13(3):e0194220. doi: 10.1371/journal.pone.0194220 (PMC5858778; doi:10.1371/journal.pone.0194220)

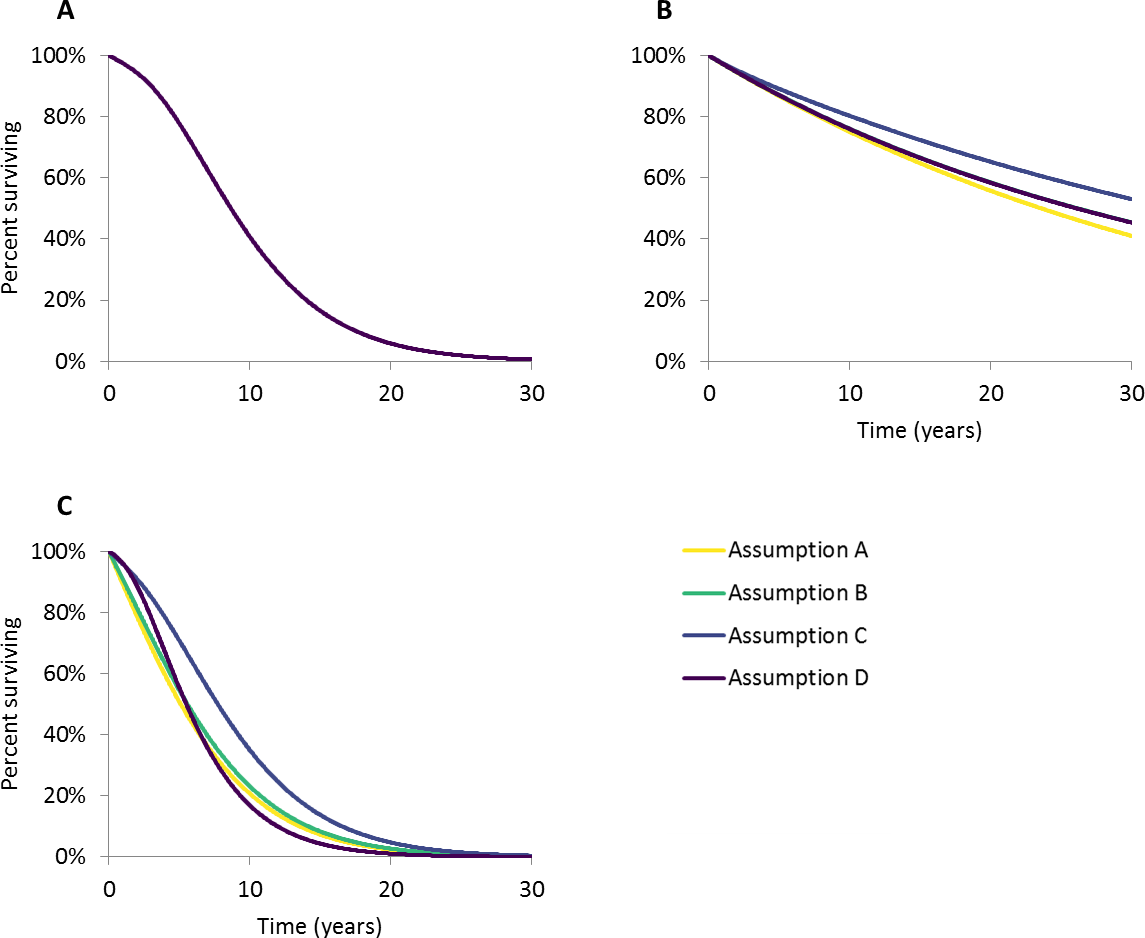

Supplement: S1 Fig — Survival curve for individuals who: A) become infected with HIV but never initiate ART; B) initiate or reinitiate ART for life; C) drop out of ART who never reinitiate treatment. (TIF) [file pone.0194220.s003.tif]

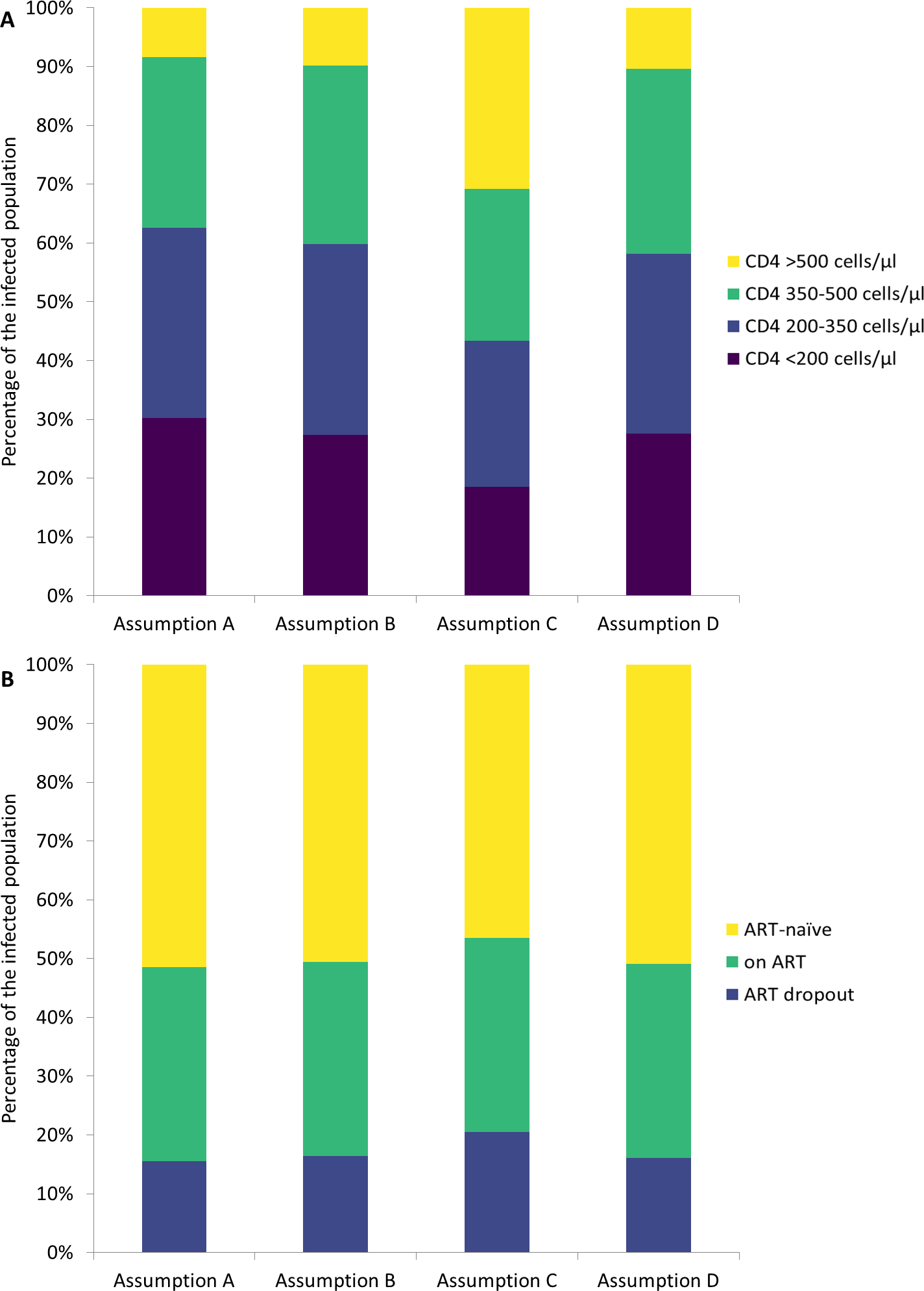

Supplement: S2 Fig — (TIF) [file pone.0194220.s004.tif]

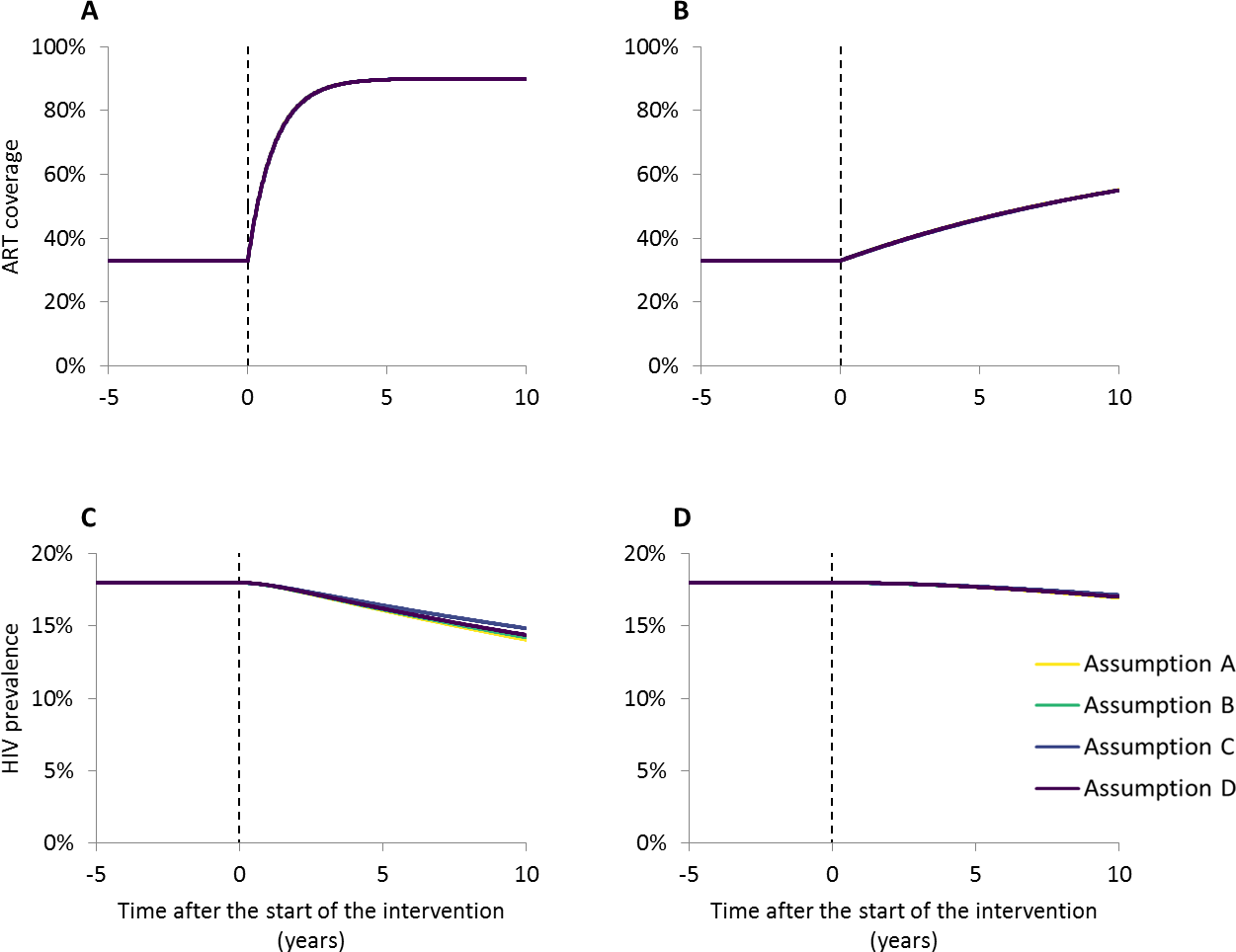

Supplement: S3 Fig — (TIF) [file pone.0194220.s005.tif]

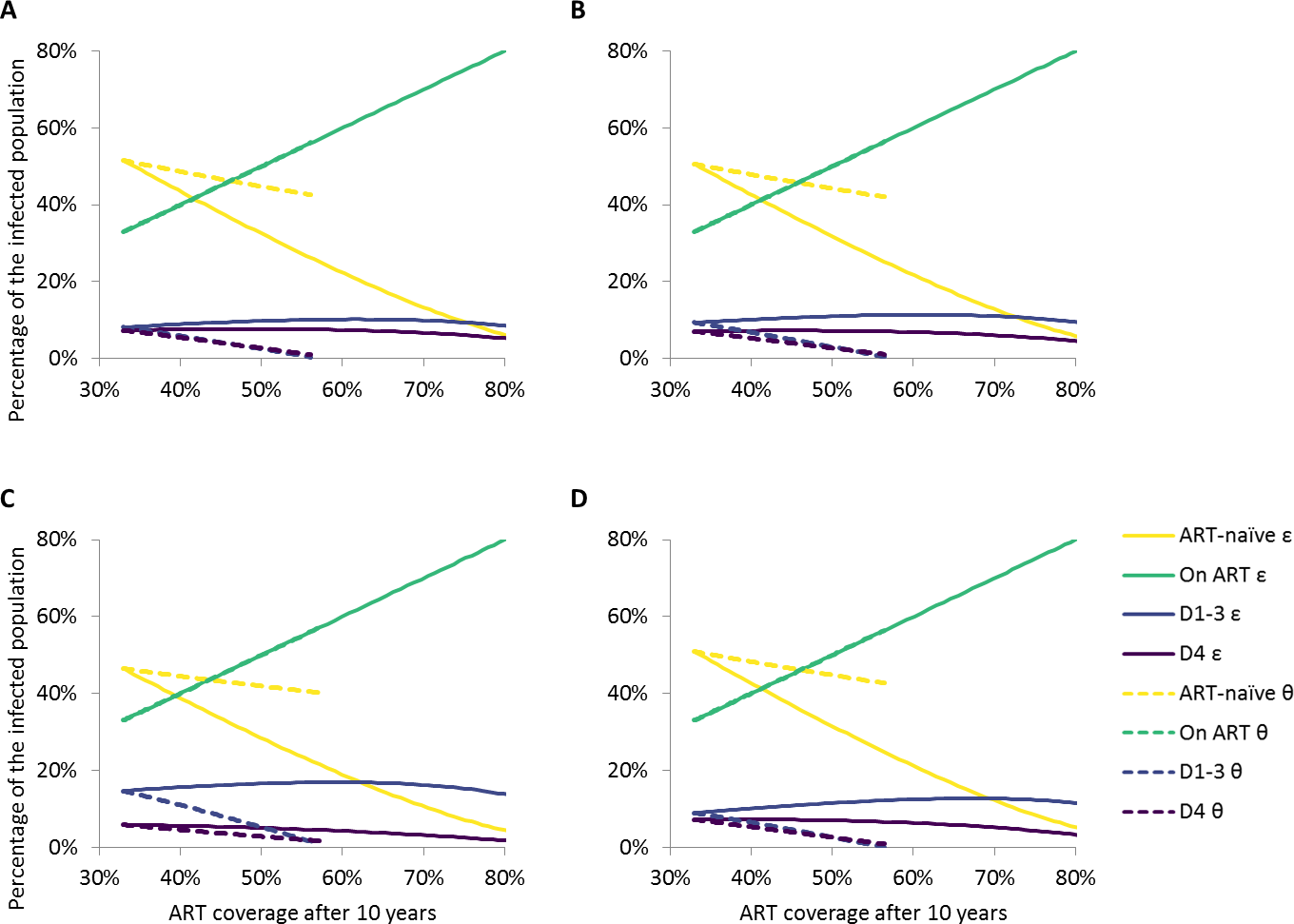

Supplement: S4 Fig — (TIF) [file pone.0194220.s006.tif]

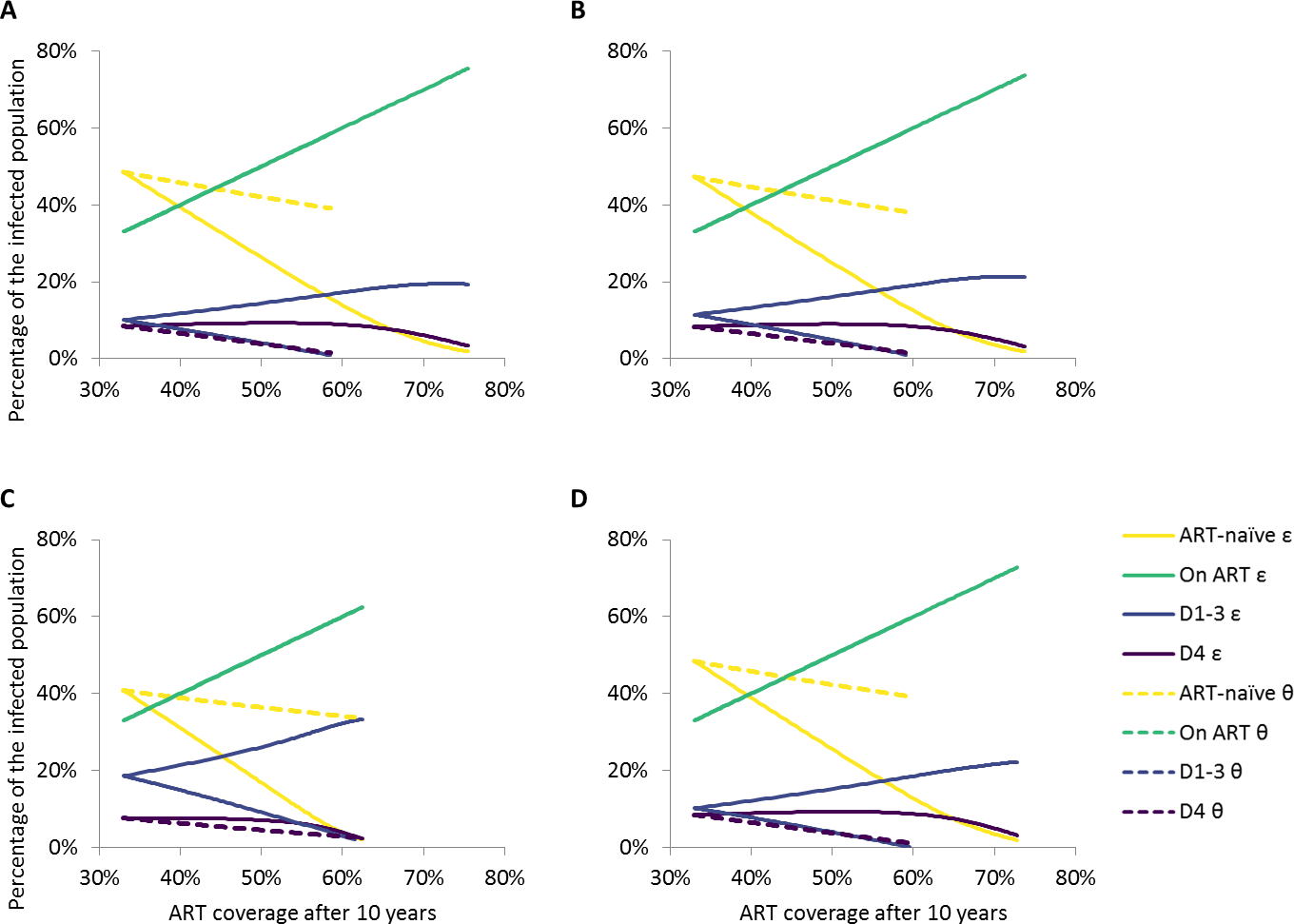

Supplement: S5 Fig — (TIF) [file pone.0194220.s007.tif]

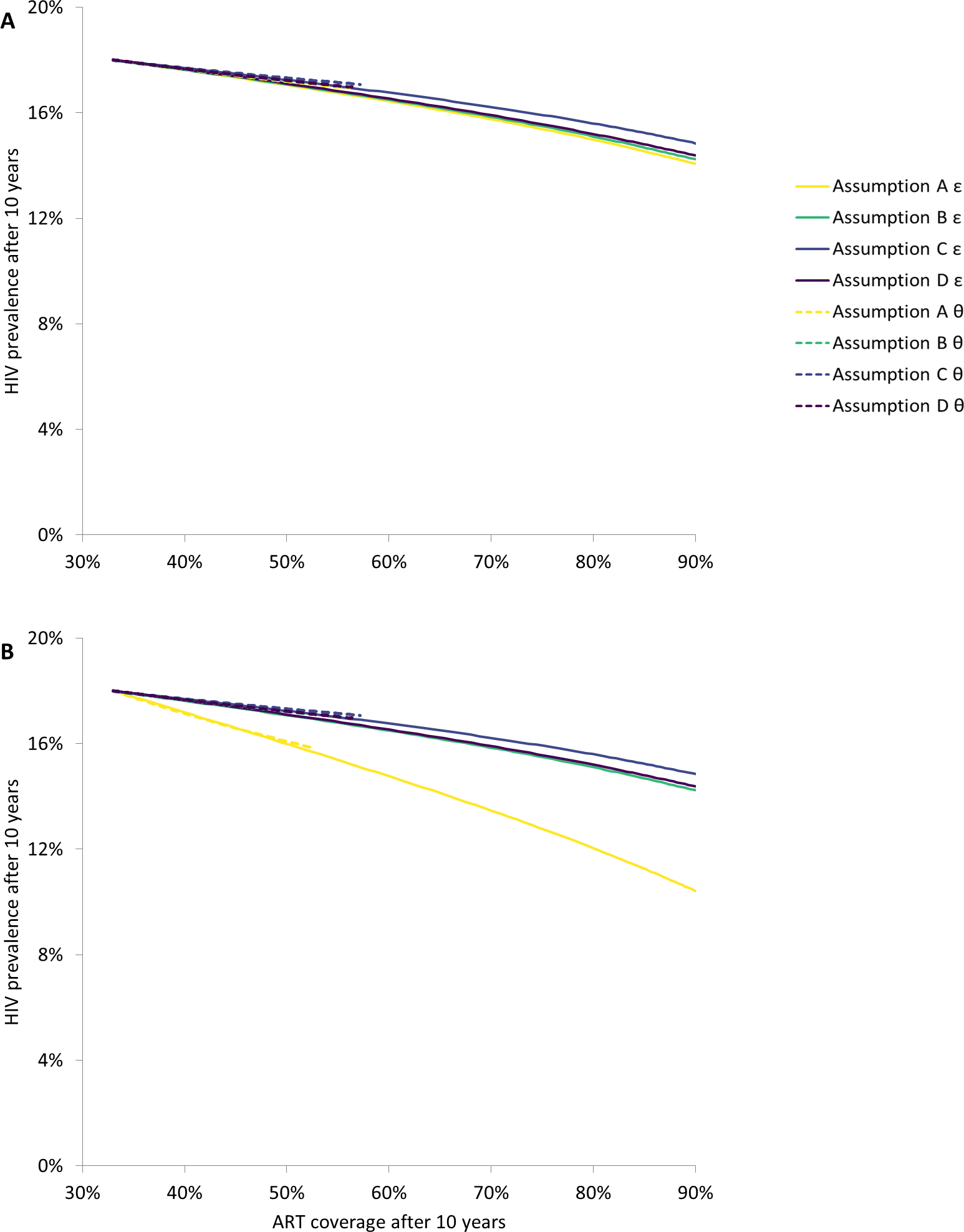

Supplement: S6 Fig — (TIF) [file pone.0194220.s008.tif]
